# Recent advances in technologies for the controlled release of aromatic compounds: From encapsulation to electrospinning

**DOI:** 10.1016/j.crfs.2026.101586

**Published:** 2026-09-29

**Authors:** Mahshid Mojarad, Zolaikha Shiravani, Elahe Abedi, Dornoush Jafarpour, Mehran Sayadi, Seyed Mohammad Bagher Hashemi

**Affiliations:** a Department of Food Science and Technology, School of Agriculture, Shiraz University, Shiraz, Iran; b Department of Food Safety and Hygiene, School of Health, Fasa University of Medical Sciences, Fasa, Iran; c Department of Food Science and Technology, Faculty of Agriculture, Fasa University, Fasa, Iran; d Department of Food Science and Technology, Fa.C., Islamic Azad University, Fasa, Iran; e Department of Food Hygiene, Faculty of Veterinary Medicine, Amol University of Special Modern Technologies (AUSMT), Amol, Iran

**Keywords:** Aromatic compounds, Encapsulation, Controlled release, Electrospinning, Electrohydrodynamic techniques, Food industry applications

## Abstract

Plant aromatic compounds are molecules with biological, antimicrobial, functional, and sensory properties that, in addition to their desirable aroma and flavor, can play an effective role in improving the quality and acceptability of products in various industries, including food, pharmaceutical, and cosmetic-healthcare industries. However, due to their low molecular weight, volatility, and sensitivity, these aromatic compounds are prone to evaporation and oxidation during processing and storage, influenced by environmental factors such as air, light, moisture, and heat. This review provides a comprehensive analysis of conventional encapsulation techniques in comparison with emerging electrohydrodynamic methods, particularly electrospinning. Electrospinning enables the fabrication of fibrous materials with high surface-area-to-volume ratios, interconnected porosity, and nanoscale features, which enhance compound protection, bioavailability, and release control. The application of this technique in food systems is promising, as it allows efficient incorporation of flavor compounds while maintaining their functional integrity. Finally, the review highlights the advantages, limitations, and future perspectives of each method, emphasizing the potential of electrospinning to drive innovation in functional foods and extend its applications to sectors such as pharmaceuticals, cosmetics, and health.

## Highlights

• Electrospinning enables controlled release of volatile flavor compounds.
• Encapsulation improves stability and shelf life of essential oils.
• Nanofibers enhance antimicrobial and antioxidant food packaging.
• Controlled release reduces cytotoxicity and preserves bioactivity.

## Graphical abstract

**Figure:**
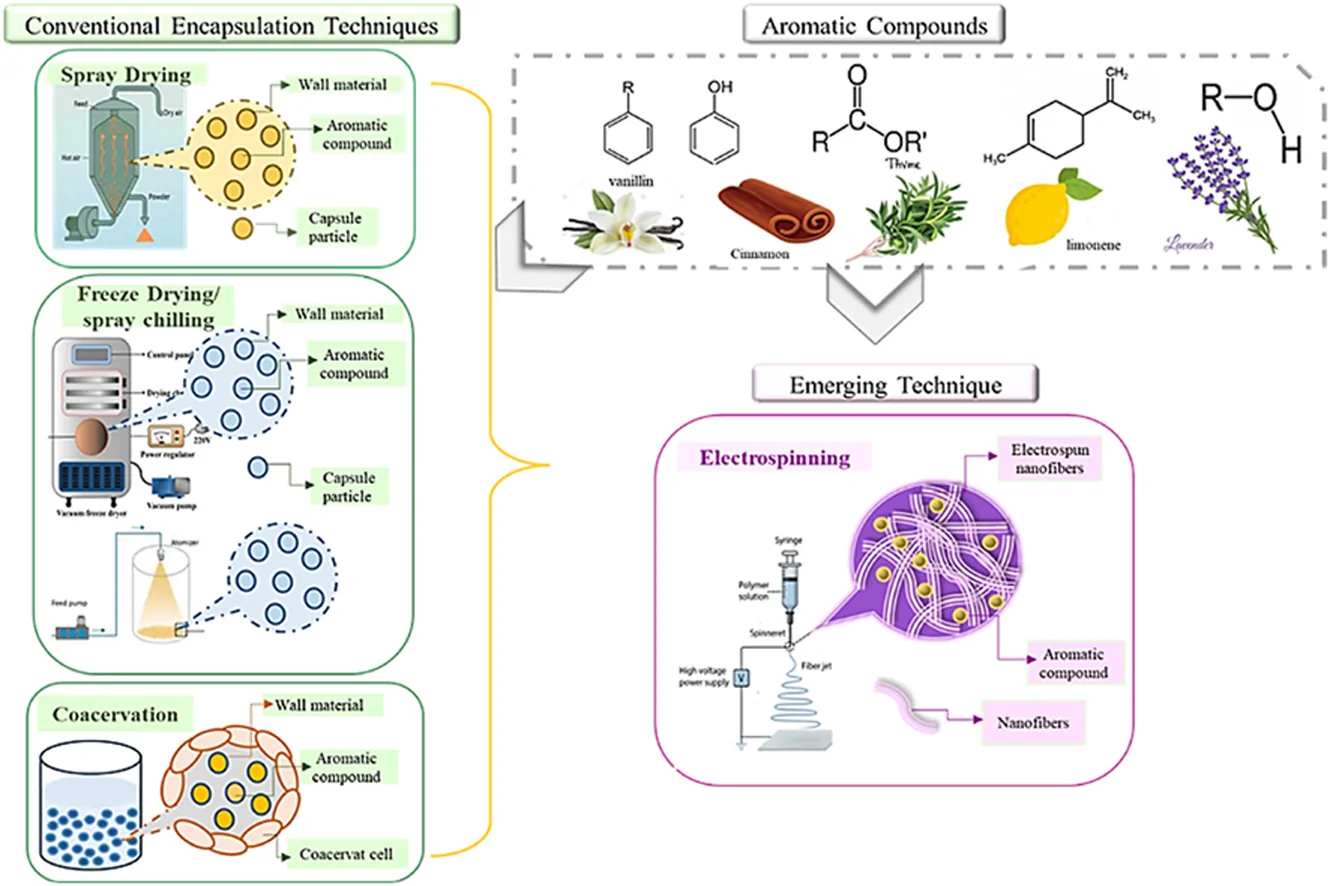

## 1 Introduction

Flavor compounds are considered one of the most important components of food formulations, as they play a fundamental role in consumer acceptance and satisfaction (Shan et al., 2025). The terms “flavor” and “taste” are closely related but distinct. Flavor encompasses the overall sensory perception of food, including taste, touch, smell, sight, and even sound, resulting from complex interactions between organic flavor compounds (Potdar et al., 2020). Aroma, on the other hand, specifically refers to the odor perceived through the olfactory system (Hossain et al., 2025). These compounds may be naturally occurring from various food and plant sources, such as carbohydrates, fats, proteins, minerals, vitamins, or essential oils extracted from plants, spices, citrus peels, or microbial fermentation products. Sensorially and chemically, these compounds include common examples, including phenolics, terpenoids, alcohols, esters, ketones, and pyrazines, which also have antioxidant, anticancer, anti-inflammatory, and immune-boosting properties (Mosia et al., 2022). In contrast, artificial flavor compounds are synthetically produced to mimic natural flavors. These compounds can produce sensory characteristics of natural flavors, including smoky, citrus, bitter, fruity, buttery, and caramel. As shown in Fig. 1, each artificial flavorant can provide a sensory perception of a natural flavor, depending on its molecular structure and chemical diversity, which is effective for choosing the appropriate encapsulation method and for interactions with food matrices (Hossain et al., 2025).

**Fig. 1:**
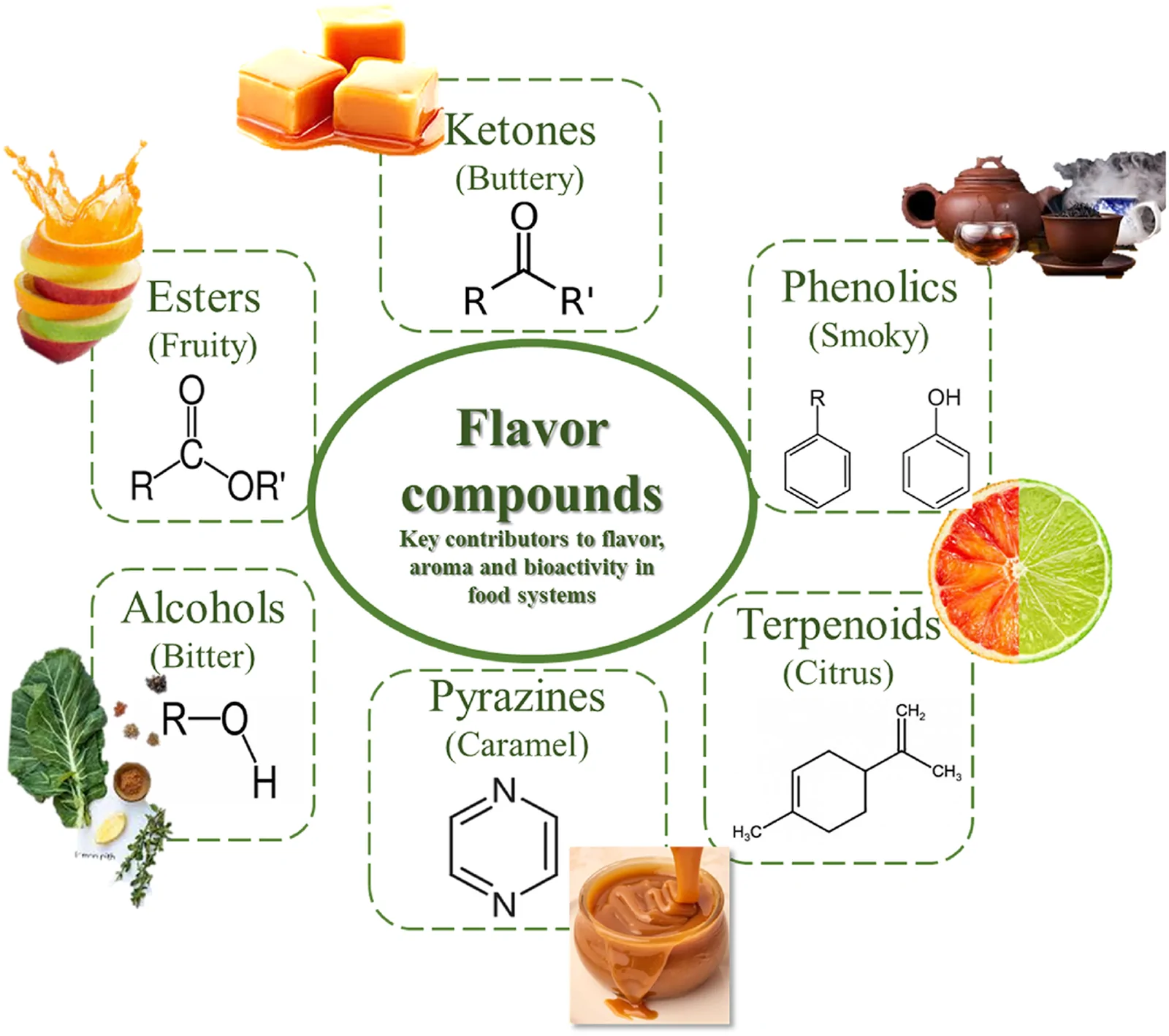
Schematic of the most common natural aromatic compounds used in the food system.

The sensory quality and taste of food are assessed based on both volatile and non-volatile components. Although over 10,000 volatile compounds have been identified in various foods, only a small fraction directly contributes to flavor perception. This is because many volatile compounds are low-molecular-weight and easily degraded during processing and storage by heat, light, evaporation, or poor hydrophilicity (Gan et al., 2023). Also, in some food products (such as cookies and bread), flavors are lost during processing (baking) or develop off-flavors. They are released when the food is placed in the mouth. Therefore, maintaining flavor stability is an important issue for food manufacturers (Shan et al., 2025).

To address these challenges, encapsulation technologies have been widely investigated as innovative methods for stabilizing volatile compounds. Encapsulation protects flavor compounds from degradation, delays their release, prevents oxidation, and improves controlled release, ultimately enhancing organoleptic quality (Kłosowska et al., 2023). This process can be implemented at macro (>5000 μm), micro (1–1000 μm), and nano (1–100 nm) scales. Encapsulation at these scales improves physicochemical stability (Sonawane et al., 2020a, Sonawane et al., 2020b). Although the microscale is more common in the food industry, recent evidence suggests that nanoencapsulation offers improved stability and controlled release, as well as enhanced solubility in liquids and penetration through biological barriers (English et al., 2023; Sonawane et al., 2020a, Sonawane et al., 2020b). To create these scales, conventional techniques such as spray drying, freeze drying, emulsification, inclusion complexation, and liposome formation are widely used; however, they can cause diffusion of flavor compounds, chemical or physical interactions, and off-flavors, which reduce consumer acceptance (Rahim et al., 2025; Muhoza et al., 2023).

Electrospinning has recently gained attention as an effective alternative for encapsulating flavor compounds. The technique is versatile and efficient, producing fibrous structures characterized by high surface-area-to-volume ratios and interconnected porosity. In contrast to conventional encapsulation methods, electrospinning operates under mild conditions, minimizing thermal and chemical degradation of volatile compounds (Wang et al., 2023).

Considering the increasing demand for herbal and natural products as well as the pivotal role of flavor and aroma in the food, pharmaceutical, and cosmetic industries, this review presents a comparative analysis of conventional and electrospinning encapsulation techniques as an innovative method and the impact of each process on the stability and controlled delivery of aromatic compounds. Furthermore, the relationships among carrier materials, principles, and mechanisms of the methods; encapsulation efficiency; release behavior; the advantages and challenges of each method; industrial applications in food systems; and the future prospects of these technologies for flavor encapsulation will be discussed.

The references used in this study were collected by searching major scientific databases, including Scopus, Web of Science, ScienceDirect, and PubMed, covering the period from 2015 to 2025. The search aimed to review recent developments in conventional encapsulation methods, including spray drying, freeze drying, spray cooling, and coacervation, as well as electrospinning-based systems for aromatic compounds, release mechanisms, and food-industry applications. The selection criteria for references focused on keywords including “encapsulation”, “electrospinning”, “aromatic compounds”, “flavor compounds”, “volatile compounds”, and “controlled release”. In addition, studies were selected based on encapsulation process type, wall material properties, release behavior, and industrial applicability to enable more meaningful comparison and analysis. Studies that lacked sufficient information or were not relevant to food systems were excluded from this review.

## 2 Definition and classification of conventional encapsulation methods

Encapsulation is a technique that involves the formation of delivery systems to preserve the chemical, physical, and biological stability of active compounds (Fig. 2) while enabling their controlled and targeted release under specific environmental conditions (Sonawane et al., 2020). Designing effective encapsulation systems requires a thorough understanding of the chemical properties of flavoring agents, the selection of suitable encapsulation techniques, and consideration of processing conditions and the product's final application (Gumfekar, 2020). In practice, volatile flavors are often transformed into oil-based formulations and stored in a powdered form using various encapsulation methods (Potdar et al., 2020). Encapsulation techniques can be broadly classified as physicochemical methods (coacervation, emulsion, supercritical fluid deposition) (Hernández-Nava et al., 2020), physicomechanical methods (spray drying, freeze drying) (Favaro-Trindade et al., 2021), chemical methods (polymerization, cross-linking) (Rojas et al., 2024), and electrohydrodynamic methods (electrospinning) (Saifullah et al., 2019). Among these methods, emulsions, spray drying, extrusion, electrospinning, and coacervation are the most widely used flavor encapsulation methods (Agriopoulou et al., 2023). The resulting capsules differ in size, morphology, release profile, and stability depending on the technique and materials used (Kłosowska et al., 2023).

**Fig. 2:**
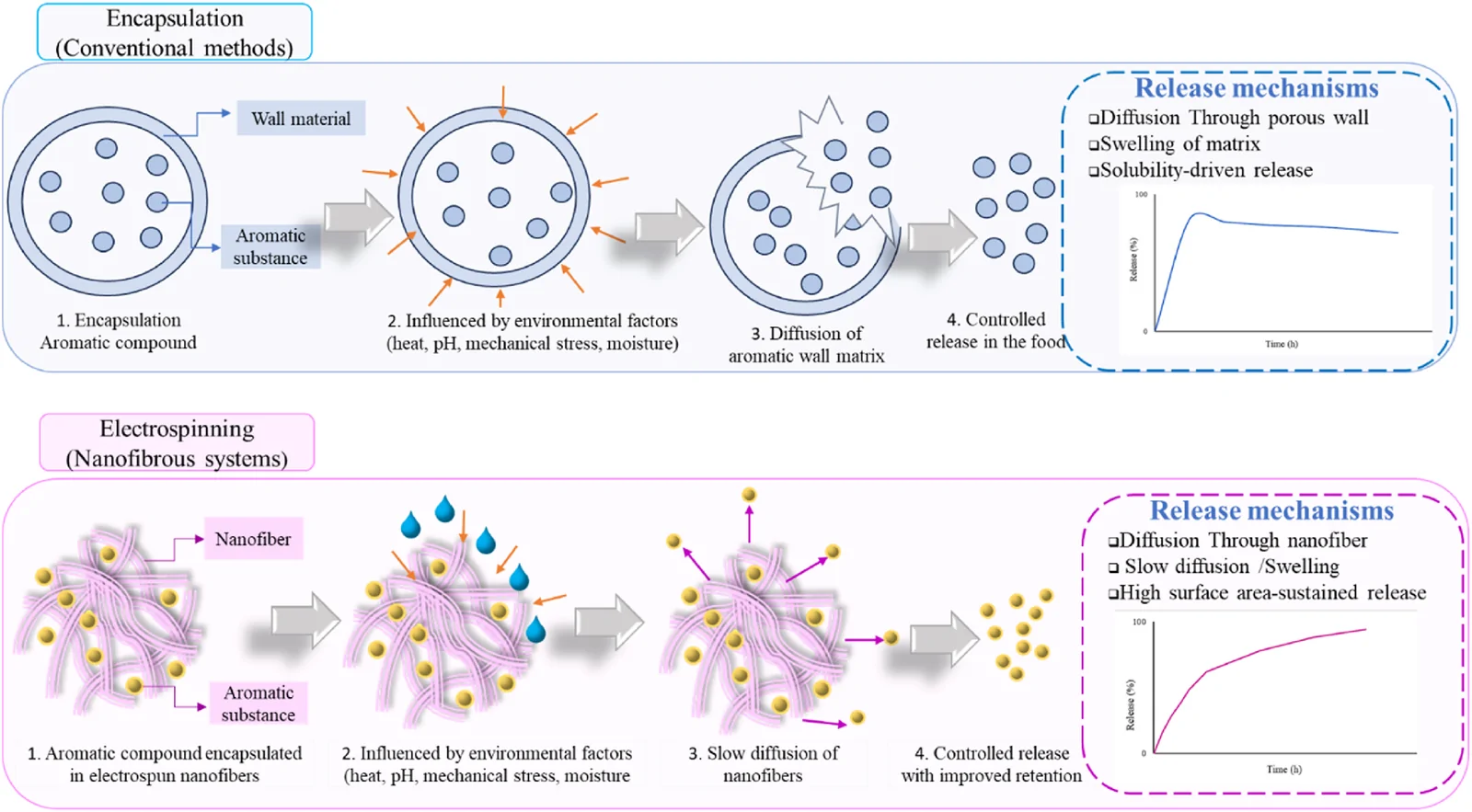
Schematic of how to release aromatic substances in the method of Encapsulation and Electrospinning.

A critical aspect of encapsulation is flavor release, which refers to the migration of flavor molecules from the encapsulated system into the food matrix or saliva at a specific rate. Release can be controlled or uncontrolled, depending on the properties of the wall material, the encapsulated flavor, particle size, molecular weight, and solubility in the food matrix. Controlled release ensures that flavor compounds are delivered at the right time and location, optimizing sensory perception and product performance (Xu et al., 2024).

In the following, three conventional encapsulation methods are introduced as common and widely used approaches for the controlled release of aromatic compounds. These methods were selected based on their widespread application, recent research developments, and relevance to food systems (Rezvankhah et al., 2020; Timilsena et al., 2019). Although conventional encapsulation technologies differ in their mechanisms and processing conditions, none is universally superior, and the selection of an appropriate method depends on the characteristics of the active compound, carrier material, and intended application (Bakry et al., 2016; Premjit et al., 2022). Spray drying remains one of the most widely applied industrial encapsulation techniques because of its simplicity, relatively low cost, scalability, and high production throughput (Fang and Bhandari, 2010; Kumar et al., 2025). Freeze drying provides improved protection for heat-sensitive compounds due to its low-temperature processing conditions; however, it is associated with longer processing times, higher energy consumption, and increased operational costs (Potdar et al., 2020; Rezvankhah et al., 2020). Coacervation can achieve high encapsulation efficiency for specific applications through interactions between wall materials and core compounds, but it often requires precise control, including formulation parameters such as pH, ionic strength, and polymer characteristics (Shaddel et al., 2018; Timilsena et al., 2019). Therefore, the choice of encapsulation method should be determined by the intended food application, the physicochemical properties of the flavor compounds, economic considerations, and production scale, rather than by encapsulation efficiency alone (Bakry et al., 2016; Premjit et al., 2022).

### 2.1 Spray-drying method

Spray drying represents one of the most commonly used and cost-effective techniques for flavor encapsulation. In this method, the core material, such as a flavor or bioactive extract, is first dissolved or dispersed within a suitable carrier matrix to form a homogeneous mixture. This mixture is then atomized into a drying chamber, where rapid evaporation of the solvent produces predominantly spherical encapsulated particles. Inert gases such as air or nitrogen are often used to prevent oxidation (Mohammed et al., 2020). For example, anthocyanins extracted from blueberries were spray-dried under varying temperature and wall-material compositions, with the optimal formulation (9% maltodextrin + 9% hi-maize at 140 °C) exhibiting maximal stability, minimal degradation, and enhanced release under simulated gastrointestinal conditions (da Rosa et al., 2019). Similarly, strawberry flavor encapsulation achieved 85% efficiency using maltodextrin, modified starch, and gum arabic at an inlet temperature of 190 °C, producing spherical, homogeneous microcapsules with ∼90% solubility (Balci-Torun and Ozdemir, 2021). Spray-drying of essential oils has also been reported as an effective method to improve encapsulation efficiency and shelf life by optimizing wall/core composition, emulsion properties, and drying parameters (Nguyen et al., 2021). The actual particle temperature during spray drying is typically lower than the air temperature due to the latent heat of evaporation and high surface-area-to-volume ratios, which allows rapid drying while minimizing thermal degradation (Mohammed et al., 2020). However, limitations include powder adhesion to equipment, loss of aroma due to thermal reactions, and variable retention of volatile compounds. While high-carbon volatiles may retain up to 95% of their aroma, low-molecular-weight volatiles (e.g., acetaldehyde) may retain as little as 20%, resulting in potential inconsistencies between liquid and powdered flavors (Mohammed et al., 2020; English et al., 2023; Sri Padma and Joshi, 2023).

Although spray drying is recognised as the most common encapsulation technology in the food industry because of its low cost, high processing speed, and suitability for continuous production, comparison of different studies shows that the performance of this method is highly dependent on the type of wall material, the core-to-wall ratio, emulsion characteristics, and processing conditions (Gharsallaoui et al., 2007). This variability in formulations and evaluation methods has led to research findings that are not always directly comparable with respect to encapsulation efficiency, particle size, stability, and retention of volatile compounds, and sometimes even contradictory results have been reported (Jafari et al., 2008). In addition, a significant proportion of the available studies have been conducted at the laboratory scale, and limited information is available regarding the behaviour of these microcapsules under real processing conditions, in complex food matrices, and during long-term storage (Fang and Bhandari, 2010). Therefore, although spray drying is considered a highly efficient industrial technology, a realistic evaluation of its performance requires the standardization of experimental protocols and the implementation of industrial-scale, long-term studies to draw more reliable conclusions regarding the superiority of one formulation or carrier system over others.

Although spray drying is recognised as the most common encapsulation technology in the food industry because of its low cost, high processing speed, and suitability for continuous production (Gharsallaoui et al., 2007), comparison of different studies shows that the performance of this method is highly dependent on the type of wall material, the core-to-wall ratio, emulsion characteristics, and processing conditions (Jafari et al., 2008). This variability in formulations and evaluation methods has led to research findings that are not always directly comparable with respect to encapsulation efficiency, particle size, stability, and retention of volatile compounds (Fang and Bhandari, 2010). In addition, a significant proportion of the available studies has been conducted at the laboratory scale, and limited information is available regarding the behaviour of these microcapsules under real processing conditions, in complex food matrices, and during long-term storage (Bakry et al., 2016).

### 2.2 Spray-Chilling and freeze-drying method

Spray chilling (also termed prilling or spray cooling) is a process comparable to spray drying; however, it uses a cooling chamber operating at 34–42 °C rather than hot air. In this method, the active compound is emulsified in a lipid-based carrier, and the resulting dispersion is solidified by cooling, forming microcapsules (Potdar et al., 2020). Suitable wall materials include fats, waxes, polyethylene, fatty acids, and fatty alcohols (de Abreu Figueiredo et al., 2022). Spray chilling offers advantages such as reduced thermal degradation, suitability for heat-sensitive flavors, and cost-effectiveness. However, particle-size control is challenging, only lipid-based carriers are useable, and flavors must remain stable at the lipid melting point, thereby limiting applications (Perinelli et al., 2020). Notable applications include coating 2-acetyl-1-pyrroline (2AP) with paraffin to improve stability (Yin and Cadwallader, 2019) and masking bitter plant extracts for incorporation into dark chocolate to enhance sensory appeal and stability (Barrientos et al., 2024). Although freeze-drying is also a suitable and highly efficient method for encapsulating heat-sensitive flavors and consists of three main steps: freezing, primary drying (sublimation), and secondary drying, this process is energy-intensive and slower than spray cooling and spray drying (Potdar et al., 2020). The dependence of spray chilling on lipid-based wall materials limits the range of compounds that can be encapsulated and may affect flavor release behaviour in aqueous food systems (Comunian and Favaro-Trindade, 2016; Rezvankhah et al., 2020). On the other hand, the high energy consumption, long processing time, and high equipment and maintenance costs of freeze-drying reduce the feasibility of using this method for large-scale food production (Potdar et al., 2020; Rezvankhah et al., 2020). Consequently, the choice between these techniques should be based not only on encapsulation efficiency but also on the physicochemical properties of the flavor compound, the intended food matrix, and the economic requirements of the manufacturing process (Bakry et al., 2016; Timilsena et al., 2019).

According to studies, this method is suitable for compounds where the preservation of phenolic content, color, aroma, and nutritional value is of great importance. Accordingly, observations on green tea have reported an improvement in the color and phenolic content characteristics of the product (Golpira et al., 2021). In addition, this method is considered one of the most expensive methods among conventional methods due to its industrial costs, which are several times higher than other methods, storage, transportation of the produced particles, and long process time. Accordingly, it is mainly used to preserve the aroma and flavor of high-value foods such as coffee and spices (Mazár et al., 2025).

In a comparative report conducted by Lee et al., (2022) on chamomile extract using different convective spray drying and freeze drying methods, it was found that freeze drying retained the total polyphenol content better. However, comparing the efficiency of convective spray drying (about 90%) and freeze drying (about 84%) showed that freeze drying is not always the optimal choice, depending on the characteristics of the core material, and that it has limited use on an industrial scale, especially in the food industry. On the other hand, freeze-drying offers advantages over spray drying, such as higher retention of volatile compounds, which reduces thermal degradation and facilitates the controlled release of aromatic compounds by creating a porous structure. However, freeze-drying has limitations, including high energy consumption, long processing times, and relatively high production costs (Rezvankhah et al., 2020).

### 2.3 Coacervation method

Coacervation relies on emulsion formation, followed by the deposition of a wall material around the droplets. Typically, wall materials are proteins or polysaccharides that adsorb around the flavorant before solidification, forming micro- or nanocapsules, depending on solute composition. This method can produce simple or complex coacervates that encapsulate both hydrophilic and hydrophobic flavors, and commonly uses gelatin as the polymer for simple coacervation (Timilsena et al., 2019). Advantages include the protection of heat-sensitive flavors and improved aroma retention. However, process control is challenging due to pH, ionic strength, polymer molecular weight, solvent, and flavor properties, which can lead to variable particle sizes, aggregation, and increased production costs. (Timilsena et al., 2019). Recent studies report successful coacervation of cinnamon extract in ice cream and flavor granules using alginate, achieving encapsulation efficiencies up to 97% and effectively masking undesirable flavors (de Souza et al., 2020; Moawad et al., 2025).

Although coacervation is widely used to encapsulate both hydrophilic and hydrophobic compounds, including flavor compounds, systematic studies investigating how the molecular characteristics of flavor compounds influence the encapsulation mechanism, phase behavior, and final capsule properties remain limited (Timilsena et al., 2019). Available evidence suggests that molecular properties such as electrical charge and hydrophobicity play critical roles in coacervate formation and stability, potentially leading to considerable variations in encapsulation efficiency and release behavior among different formulations (Schmitt and Turgeon, 2011; Timilsena et al., 2019). Therefore, the development of predictive models and mechanistic studies to better understand interactions between flavor compounds and wall polymers remains an important research priority to improve the reproducibility and industrial applicability of coacervation-based encapsulation systems (Timilsena et al., 2019). Conventional encapsulation techniques are widely used for their simplicity and cost-effectiveness, yet challenges such as high process temperatures, variable particle size, limited carrier materials, and inconsistent encapsulation efficiency have prompted interest in electrospinning as a promising alternative for aroma and flavor encapsulation (English et al., 2023; Mohammed et al., 2020; Potdar et al., 2020).

### 2.4 Selection of wall materials based on conventional methods of encapsulating aromatic compounds

As shown in Table 1, the performance of wall materials depends not only on their physicochemical characteristics but also on the encapsulation technique and the properties of the aromatic compounds. No single wall material is universally suitable for all applications.

**Table 1:** Comparative characteristics of wall materials used in the most common techniques and Electrospinning.

Wall Material	Encapsulated Compound	Drying Method	Research Objective	Specific Benefits	General properties	References
Maltodextrin	Asparagus	Spray-drying	Improving the retention of volatile compound (1-octen-3-ol) in asparagus by the effect of maltodextrin content and encapsulation method	- Increasing the amount of maltodextrin (up to 67%) increased the retention of important asparagus compounds (1-octen-3-ol) - Drying conditions increased the glass transition temperature and higher retention of volatile compounds - Particle size distribution was more uniform with the type of wall material	Reducing the final moisture content of the product, creating smaller and more spherical particles, and increasing the water reabsorption capacity	(Siccama et al., 2021)
Gum Arabic	Ginger	Coacervates	Release control and increased stability while maintaining the properties of ginger oil	- Ginger encapsulated by this method has better physicochemical properties than free oil. - The homogeneous particle size and viscous state of the coating made the ginger release more efficiently.	Good heat protection from volatile compounds, high solubility in water, and high emulsifying properties for these techniques	(Tavares et al., 2021)
Maltodextrin + Gum Arabic	Fruit oil	Spray drying & Freeze drying	Effect of encapsulation methods on core coating materials containing fruit oils	- The freeze-drying method preserved the aromatic compounds of the fruit oils better. - However, due to the coating materials, the spray-drying method produced more uniform and stable particles. - The wall materials and encapsulation method should be selected based on the research objective.	The choice of wall material for each method affects the shelf life of aromatic compounds. Freeze-dryers are suitable for heat-sensitive compounds. Spray dryers create more uniform particles with less moisture.	(Yaman et al., 2023)
Whey Protein Isolate	Thyme	Freeze drying	The effect of whey proteins for encapsulating thyme essential oil against heat, improving release, and stability	Using the freeze-drying method for whey protein increases quality and shelf life. Due to the lower moisture content of the particles, the release of thyme essential oil from the particles is more controlled, and the encapsulation efficiency is improved by about 100%.	Application for heat-sensitive materials & can be used in the freeze-drying technique due to the sensitivity of its surface proteins, The modification of bioactive release properties, & Production of uniform and flat fibers	(Volić et al., 2022)
Zein	Clove	Electrospinning	Modeling the gradual release of clove oil from zein nanoparticles and increasing its shelf life	- Reduced and consistent release rate with higher mechanical strength - Increased shelf life of clove oil in nanofibers due to better moisture barrier	Improved morphological characteristics, controlled release	(Alinaqi et al., 2021)
Chitosan		Electrospinning, Coacervates, Spray drying	Study of encapsulation of aromatic essential oils with antibacterial properties (thyme, clove, cinnamon, etc.) in chitosan nanoparticles to evaluate the release, stability, and antibacterial properties of these essential oils	- Particle formation of about 100-300 nm depending on the type of essential oil and the encapsulation method - Encapsulation efficiency above 70%, especially for thyme and clove oils with the electrospinning method - Gradual and long-term release in the coacervation method - Increased antibacterial properties and higher shelf life in all methods, especially electrospinning and spray drying	Smooth surfaces and low defects with particle diameters less than 500 nm, High thermal stability & most commonly used for oily aromatic compounds due to their insolubility in water.	(Negi and Kesari, 2022)
Soy Protein	Cinnamon & Zataria	Electrospinning	Fabrication of nanofibers containing antibacterial oils for food packaging and medical adhesive applications	- Production of nanofibers with a diameter of 150-300 nm - Increase in viscosity due to the presence of soy proteins - The effect of these nanofibers with sustained and appropriate release on *Staphylococcus aureus* and *Escherichia coli* bacteria	The modification of bioactive release properties & beaded fibers	(Raeisi et al., 2023)
β-cyclodextrin (β-CD)	Cinnamon	Electrospinning	Encapsulating cinnamon oil with this wall material to increase its resistance in high-humidity applications and effective release to utilize its antioxidant properties while preserving aroma and flavor compounds	- Reducing the volatility of aromatic compounds in cinnamon essential oil and increasing its stability. - The coating effect of the wall material and the choice of the electrospinning method for this oil have increased its resistance to moisture. - Also, by creating uniform and thin particles, it can be effective in increasing its antioxidant properties.	Improving the strength and textural properties of fibers and producing transparent fibers & protects aromatic compounds from temperature and pH fluctuations	(Guan et al., 2024)

Polysaccharides are generally preferred for their excellent film-forming ability and suitable physicochemical properties, whereas proteins often offer higher emulsifying capacity and encapsulation efficiency (Calderón-Oliver and Ponce-Alquicira, 2022; Timilsena et al., 2020). Consequently, selecting an appropriate wall material requires balancing encapsulation performance, compatibility with the core material, processing conditions, and economic considerations (Calderón-Oliver and Ponce-Alquicira, 2022). These findings demonstrate that optimization of wall materials is as important as the encapsulation technology itself for achieving efficient controlled-release systems. The performance and selection of wall materials play an important role in determining the microencapsulation efficiency, mechanical integrity, and overall shelf life of encapsulated systems (Đorđević et al., 2015). The most commonly used wall materials include polysaccharides (such as starch, chitosan, pectin, and natural gums) and proteins of animal or plant origin (such as whey protein, caseinate, gelatin, and soy protein) (Timilsena et al., 2020). Polysaccharides can often serve as coatings to extend the shelf life of volatile compounds due to their physicochemical properties, such as viscosity, solubility, and strong film-forming ability, while proteins can achieve higher microencapsulation efficiency due to their strong emulsifying properties during matrix formation (Calderón-Oliver and Ponce-Alquicira, 2022; Timilsena et al., 2020). However, compatibility between the wall materials and the chemical nature of the volatile compounds is a fundamental principle for preserving and controlling their release, which can affect the stability and sensory quality of the aromatic compounds (Schmitt and Turgeon, 2011). Polysaccharides such as maltodextrin and gum arabic, and proteins like whey protein and caseinate, are widely used in conventional encapsulation of aromatic compounds (Díaz-Montes, 2023; Timilsena et al., 2020; Xiao et al., 2022). Maltodextrin, despite its high solubility, has limited emulsifying capacity. Gum arabic provides excellent protection against the loss of volatile compounds, whereas whey protein and caseinate offer good protection against oxidation and favorable functional properties; however, their cost and interactions with core materials may limit their application (Díaz-Montes, 2023; Malamatari et al., 2020; Timilsena et al., 2020; Xiao et al., 2022).

#### 2.4.1 Wall materials in spray drying

The choice of encapsulating agents in spray drying depends on key carrier characteristics, including solubility, viscosity, emulsification performance, and film-forming properties. Polysaccharides are frequently favored because of their high loading capacity, excellent solubility, and relatively low viscosity (Sharif et al., 2020). For example, maltodextrin is commonly used to coat aromatic compounds, while excessive dextrose may reduce efficiency due to powder adhesion. Optimal conditions, such as 30% maltodextrin, have shown the best performance in preserving flavor (84%) (Li et al., 2020). In a study encapsulating lavender and peppermint oils at 170 °C, gum arabic alone was insufficient to form a stable coating; the addition of maltodextrin improved thermal protection and particle morphology. These findings highlight that the type and concentration of wall and core materials significantly affect encapsulation performance, particle morphology, and stability (Pilicheva et al., 2021). According to English et al. (2023), this method is applicable when the wall material has high water solubility, low viscosity, and high emulsifying properties. Otherwise, the high processing temperature in this method may accelerate the degradation and evaporation of aromatic compounds (English et al., 2023). Therefore, appropriate wall materials can increase encapsulation efficiency and improve the stability of volatile compounds, depending on their composition. In general, spray drying offers lower operating costs than other methods but lower encapsulation efficiency due to heat, which may not be suitable for heat-sensitive compounds in industry. However, the choice of spray drying largely depends on the balance between selecting economically suitable wall materials and preserving aromatic compounds.

#### 2.4.2 Wall materials in freeze drying and Spray-Chilling

Freeze drying (lyophilization) is a dehydration process based on sublimation, in which water is removed directly from ice without passing through the liquid phase. Compared with spray drying, freeze-drying produces powders with distinct particle-size distributions and characteristic morphological structures, often preserving structural integrity more effectively (Rezvankhah et al., 2020). Polysaccharides such as maltodextrin, often combined with pectin or soy protein isolate, provide desirable physical properties for encapsulating aromatic compounds; however, the open, porous structure of freeze-dried powders can increase sensitivity to oxidation and lead to uncontrolled release during storage (Souza et al., 2017). For example, hop phenolics were freeze-dried using maltodextrin, gum arabic, and their mixtures. Gum arabic provided the highest yield and coverage of bitter acids, while maltodextrin enhanced encapsulation efficiency and storage stability, demonstrating the importance of tailoring wall-material composition to core properties. They reported that the physicochemical properties of the wall materials and their interactions with the core compounds play a role in determining encapsulation performance, and that the optimization and selection of wall materials are essential for protecting volatiles and controlling the release of aromatic compounds during storage (Tatasciore et al., 2023). Another study also used lipid wall materials for their ability to protect aromatic compounds from oxygen and moisture during Spray-Chilling. They reported that, unlike carbohydrate or protein matrices, lipid carriers can form hydrophobic interactions with aromatic compounds, thereby increasing shelf life and reducing premature release. Thus, they confirmed its potential as an alternative encapsulation technology for aromatic compounds (Favaro-Trindade et al., 2021). Freeze-drying is considered a very effective encapsulation approach for preserving aromatic compounds due to its low-temperature processing conditions. It offers advantages over spray-drying, such as greater retention of volatile compounds, which reduces thermal degradation, and it facilitates the controlled release of aromatic compounds by creating a porous structure. Thereby, freeze drying and spray chilling provide valuable alternatives for encapsulating heat-sensitive aromatic compounds because they minimize thermal degradation and improve the retention of volatile constituents. However, the two techniques differ considerably in their practical applicability. Freeze-drying offers excellent preservation efficiency but is limited by long processing times, high energy consumption, and production costs, whereas spray chilling is economically attractive but is restricted by the requirement for lipid-based carrier systems. Consequently, the selection of either technique should be guided by the characteristics of the target compound, the desired release behavior, and manufacturing constraints, rather than by encapsulation efficiency alone.

#### 2.4.3 Wall materials in coacervation

Coacervation produces spherical aggregates by depositing a hydrocolloid shell around the core. Common wall materials include gelatin and gum arabic, which are effective in reducing flavor loss during processing and storage (Shaddel et al., 2018). The choice of wall material is critical, as coacervate microcapsules are sensitive to pH and ionic strength, which may limit applicability to certain flavor compounds (Mohammadalinejhad and Kurek, 2021). For instance, citral essential oil (from citrus) was encapsulated using gelatin via a simple coacervation method; the resulting microcapsules enhanced stability, reduced volatility, and extended the shelf life of peaches. Treated peaches exhibited improved appearance, texture, soluble solids, acidity, and microbial stability compared to controls, demonstrating the potential of coacervation for both food and pharmaceutical applications (Z. Li et al., 2022). Another study also used this method to prepare aromatic beads with alginate and described the preservation of flavor and structure of aromatic compounds using gas chromatography-mass spectrometry (GC-MS). They reported that the encapsulation efficiency was 97% and the microcapsules had a mononuclear structure with a particle size of less than 1000 μm and a spherical shape (Moawad et al., 2025). Accordingly, this method can affect the ultimate stability and release behavior of aromatic compounds due to electrostatic interactions between wall materials and aromatic compounds (Schmitt and Turgeon, 2011). However, its performance is highly dependent on pH, ionic strength, and the choice of wall material in the formulation, which may make optimizing this process for industrial applications a major challenge (Timilsena et al., 2020).

Therefore, conventional encapsulation methods, such as spray drying, freeze-drying, and coacervation, are widely used and appreciated for their cost-effectiveness, simplicity, and potential to enhance the stability and protection of aromatic compounds (Premjit et al., 2022). However, these methods face challenges, including high processing temperatures, difficulty controlling particle size and morphology, limited compatibility with wall materials, and variability in encapsulation efficiency (English et al., 2023). Electrospinning has emerged as a promising approach for encapsulating aromatic compounds, offering enhanced control over particle size, morphology, and release kinetics, as well as the ability to work under mild processing conditions (Castro-Muñoz et al., 2023; Falsafi et al., 2023; Ghorani and Tucker, 2015; Muthukrishnan, 2022). Overall, the comparison presented in Table 1 indicates that conventional encapsulation methods remain highly relevant for industrial applications despite the emergence of electrospinning. Spray drying remains the preferred technology for large-scale food production due to its low operating costs and established industrial infrastructure (Díaz-Montes, 2023). Freeze-drying is more appropriate for preserving highly volatile and thermolabile aromatic compounds when product quality is prioritized over production cost (Rezvankhah et al., 2020). Coacervation is advantageous when high encapsulation efficiency and tailored release behavior are required, although its industrial implementation is limited by the need for precise formulation control (Timilsena et al., 2020).

## 3 Electrospinning as a novel encapsulation method

Electrospinning is an advanced electrohydrodynamic technique. The method produces fibers with uniform morphology and low polydispersity, making it particularly suitable for protecting fragile aromatic and bioactive compounds (Fig. 2). The resulting fibers typically have diameters ranging from nanometers to micrometers, depending on the solution properties and electrospinning parameters (Saifullah et al., 2019). Fiber formation occurs when a polymer solution containing encapsulated aromatic compounds is subjected to a high-voltage electrostatic field. Under these conditions, the solution is stretched into elongated, filamentous structures rather than forming spherical particles. This unique morphology enhances the stability of the encapsulated compounds and provides improved control over release kinetics (Stoyanova et al., 2023).

Advantages of electrospun fibers, electrospun nanofibers offer several advantages over conventional encapsulation methods: 1) Nanoscale fiber diameters provide a high surface area-to-volume ratio, enhancing the stability and bioavailability of encapsulated compounds. 2) Porosity and fiber morphology facilitate controlled and sustained release of aromatic compounds. 3) The process avoids high temperatures, pressure, or harsh chemicals, preserving the structural and sensory integrity of flavors. 4) Fibers can be designed with tailored wettability, solubility, and interfacial properties, which influence encapsulation efficiency and release kinetics. 5) Electrospun fibers can be directly incorporated into functional foods, nutraceuticals, or controlled-release flavor systems, enabling innovative sensory and bioactive applications (Coelho et al., 2021).

Electrospinning techniques for flavor encapsulation, two common electrospinning approaches are emulsion electrospinning and coaxial electrospinning, both of which help prevent burst release and enable sustained flavor delivery (Muthukrishnan, 2022).

(1) Emulsion electrospinning is based on W/O or O/W emulsions and is especially applicable for hydrophilic flavor compounds or actives that are poorly compatible with hydrophobic polymers. Aromatic compounds are entrapped within the core of core–shell-structured fibers, thereby facilitating sustained and controlled release. The use of distinct solvents for the polymer and active phase prevents premature interaction and phase mixing (Muthukrishnan, 2022).
(2) Coaxial electrospinning involves the use of two concentric nozzles to produce core–sheath structured fibers with enhanced morphological control. The formation of a stable core–shell system depends on solution miscibility, interfacial tension, and solvent diffusion dynamics. This method is especially effective for encapsulating volatile or thermolabile aromatic compounds, as it enables fine-tuning of release behavior (Muthukrishnan, 2022).

Recent studies have demonstrated that regulating the core-shell structure can significantly improve the encapsulation efficiency of aromatic compounds and minimize the burst release of volatile compounds. This is because, in core-shell electrospinning, the active compounds are physically confined to the inner core and surrounded by an outer polymer layer, unlike in monolithic fibers. Consequently, the direct contact of volatile compounds with the external environment is reduced, and their rapid diffusion towards the fiber surface is limited. Furthermore, the release kinetics can be precisely controlled by adjusting the shell thickness, core-to-shell flow ratio, polymer hydrophobicity, and interfacial compatibility between the two layers. Increasing the shell thickness generally prolongs the diffusion pathway and reduces the initial burst release, ultimately resulting in a slower and more sustained release profile (Yang et al., 2024; Ying and Zheng, 2026). This technique is highly flexible and tunable, making it a promising platform for the food, pharmaceutical, and cosmetic sectors, especially for developing functional systems containing flavors and bioactive compounds. However, its applications face challenges in scalability, low production capacity, limited availability of solvents suitable for the food and pharmaceutical industries, and sensitivity to process parameters (voltage, viscosity, etc.).

### 3.1 Food-grade nanofibers as carriers of plant extracts

Biopolymer-based encapsulation is an effective strategy to enhance the functional quality and stability of natural ingredients in food and beverage products. According to Table 2, food-grade biopolymers exhibit distinct advantages and limitations depending on their molecular structure and processing behavior. Although natural polysaccharides provide excellent biocompatibility and sustainability, many require blending with proteins or synthetic polymers to achieve sufficient electrospinnability and mechanical stability (Z. Lin et al., 2023). Similarly, protein-based polymers generally improve fiber formation and encapsulation efficiency but may introduce challenges related to moisture sensitivity and processing conditions (Falsafi et al., 2023). These observations highlight that successful electrospinning depends not only on polymer selection but also on achieving an appropriate balance between food safety, fiber morphology, encapsulation performance, and industrial processability (Falsafi et al., 2023; Z. Lin et al., 2023). The most common natural biopolymers used in nanofiber production for bioactive and aromatic compounds include polysaccharides and polypeptides, which offer advantages such as biocompatibility, stability, and wide availability for food applications (Falsafi et al., 2023; Z. Lin et al., 2023; Stoyanova et al., 2023).

**Table 2:** Summary of studies of aromatic compounds in electrospun polymer matrices.

Encapsulated compound	Polymer matrix	Electrospinning method	Main findings	Application	Reference
Thyme	Pullulan and whey protein	coaxial electrospinning	EE^a^ = 87%, an increase in the stability (under light and temperature) of thymol, released in the small intestine.	food and pharmaceutical	Ali et al. (2024)
clove	Starch	Emulsion electrospraying	EE = 90%, reduced the water vapor permeability, and increased the tensile strength.	edible coating and bio-preservative in perishable food products	Alinaqi et al. (2021)
olive leaf	Almond gum/gelatin	electrospun nanofibers	EE = 90%, morphology with a uniform structure, enabling controlled release, masking The undesirable taste of polyphenols.	Food and pharmaceutical industries	Geravand et al. (2025)
lavender	Polylactic acid	Solution electrospinning	EE = NR, nanofibers showed bead-free morphology, and were hydrophobic with satisfactory thermal properties.	Active packaging, food coating, and wound dressing	Ismaili et al. (2024)
Saffron	Gelatin	electrospinning and freeze drying	EE = 96%, electrospinning is a better and more effective technique than freeze drying, smooth, bead-free, and homogeneously distributed morphology, Increased durability at high temperatures.	used in different foods such as desserts, dairy products, chewing gums, beverages, and tea bags	Golpira et al. (2021)
limonene	Poly (lactic acid)	Electrospun fibres	EE = NR, Evaluation of physical properties during aging of electrospun fibers containing aromatic compounds.	Production of materials with biomedical applications and food packaging.	Williams et al. (2024)
vanillin	Chitosan/poly (ethylene oxide)	Emulsion electrospinning nanofibres	EE = -NR. These fibers offered high tensile strength, thermal stability, and moisture absorption.	active food packaging	Andreica et al. (2023)
Thyme	Potato starch	Single-fluid electrospinning	EE = 99%, presented a homogeneous morphology, improved phenol retention, and high thermal stability.	food products or food packaging	Fonseca et al. (2020)
*Zataria multiflora* & Cinnamon	Soy Protein Isolate/Gelatin	Multilayer electrospinning	EE = NR, The nanofibrous film showed antimicrobial activity against Gram-negative and Gram-positive pathogens.	active food packaging	Raeisi et al. (2021)

**NR:** not reported.

a **EE** = Encapsulation Efficiency.

#### 3.1.1 Solvent systems for food-grade electrospinning

The selection of a suitable solvent system is an important factor in the production of electrospun nanofibers for food applications. Fluorinated solvents such as hexafluoroisopropanol (HFIP) and trifluoroethanol (TFE), chloroform, dichloromethane, N, N-dimethylformamide (DMF), N, N-dimethylacetamide (DMAc), N-methyl-2-pyrrolidone (NMP), tetrahydrofuran (THF), toluene, trifluoroacetic acid (TFA), have limited their application in food systems due to their toxicity, high cost, and concerns about solvent residue in the final product. Consequently, increasing attention has been directed toward green solvent alternatives, including water, acetic acid, supercritical fluids (SCFs), ionic liquids (ILs), and deep eutectic solvents (DESs) (Negi et al., 2024). However, each of these alternatives presents certain limitations. Water has a limited ability to dissolve or extract nonpolar compounds, whereas supercritical fluids, such as carbon dioxide, are generally unsuitable for the solubilization of many polar compounds. Moreover, most synthetic ionic liquids are petroleum-derived and have limited applicability in the food industry because their use has not yet been specifically regulated by the Food and Drug Administration (FDA), Codex Alimentarius, or European Union legislation (Martins et al., 2017; Mišan et al., 2020). In contrast, acetic acid is considered a relatively safe solvent that can dissolve biopolymers such as zein, gelatin, and chitosan, enabling the production of uniform nanofibers while reducing the environmental and health concerns associated with fluorinated solvents (Sun et al., 2019). In recent years, deep eutectic solvents, particularly natural deep eutectic solvents (NADESs), have emerged as promising alternatives for food applications because of their biodegradability, low toxicity, and ability to solubilize both polar and nonpolar compounds. In addition, NADES can provide a stable environment for oxidation-sensitive compounds and are therefore a good choice for encapsulating aromatic and bioactive compounds (Feng et al., 2018). Despite these advantages, using these solvents remains challenging because their physicochemical properties directly influence polymer chain entanglement, solution viscosity, conductivity, and solvent evaporation rate, ultimately affecting fiber morphology and electrospinnability. Therefore, the development of food-grade solvent systems that simultaneously ensure safety, stability, and high encapsulation performance remains a major research priority in the field of food electrospinning. Although extensive research has examined fiber morphology, mechanical properties, and cellular responses, limited attention has been paid to quantitatively assessing residual solvents in electrospun fibers and their associated toxicological concerns.

#### 3.1.2 Residual solvent analysis and safety

Gas chromatography–mass spectrometry (GC–MS) is considered a suitable analytical technique for the sensitive detection and quantification of volatile residual solvents originating from the electrospinning process, even at trace levels (Starowicz, 2021). Furthermore, high-performance liquid chromatography (HPLC) can be employed to identify and quantify non-volatile residues, particularly additives introduced during post-processing steps that may remain incorporated into the final product (Cortés-Herrera et al., 2018). Thereby, the safety assessment of electrospun food-grade fibers should include the application of analytical techniques such as GC-MS, HPLC, liquid chromatography–high-resolution mass spectrometry (LC-HRMS), Fourier transform infrared spectroscopy (FTIR), Raman spectroscopy, and NIR to identify residual solvents and degradation products, together with repeated-dose oral toxicity studies to evaluate potential systemic toxicity associated with long-term consumption (da Silva and Silva, 2026). The commercial success of nano-enabled food systems largely depends on consumer confidence and perceived safety. Concerns about component migration and limited understanding of long-term effects may hinder market acceptance. Consequently, robust safety assessment and effective communication are critical for their wider implementation.

#### 3.1.3 Food-grade polymers for electrospinning applications

**Polysaccharides** tend to form strong hydrogen bonds, which lead to high solution viscosity or gel formation (Wen et al., 2017). Carbohydrate-based fibrous systems are characterized by high thermal stability, tunable chemical functionality, and favorable interactions with aromatic compounds relative to lipid- and protein-based systems. These attributes have facilitated their increased application as versatile carriers in diverse food-related applications (Han et al., 2022). A range of polysaccharides, including cellulose, chitosan, pullulan, dextran, starch, alginate, and pectin, have been extensively applied for the encapsulation of aromatic compounds, owing to their capacity to mask undesirable sensory attributes and improve overall product quality (Han et al., 2022). Chitin and chitosan are biocompatible polymers with inherent antibacterial properties, which make them promising candidates for food-grade composite nanofiber fabrication. Chitin is a naturally synthesized biopolymer in living organisms and is converted to chitosan by solubilization under acidic aqueous conditions (Han et al., 2022). A study investigated starch-based electrospun nanofibers prepared with 50% (w/v) starch in 75% (v/v) formic acid to encapsulate thyme essential oil (TEO), a natural antioxidant. The nanofibers exhibited a 99% encapsulation efficiency and effectively protected TEO phenolic constituents, highlighting their potential for use in food products and packaging (Fonseca et al., 2020). In another study, patchouli essential oil was encapsulated in carboxymethyl cellulose–gelatin (CMC–GE) nanofibers via electrospinning. The resulting system was subsequently evaluated for its ability to prolong the shelf life of freshwater shrimp during refrigerated storage, demonstrating its potential as an active preservation strategy (Shahbazi et al., 2021). Likewise, peppermint essential oil encapsulated via electrospinning exhibited decreased water vapor permeability, reduced swelling index, and lower microbial load, along with improved physicochemical stability relative to the control, resulting in better overall acceptance during storage (Shahbazi et al., 2021). In a first-time application, electrospun polyvinyl alcohol (PVA)/fucoidan (FUC) nanofibers were used to encapsulate the probiotic *Lactiplantibacillus plantarum* 69-2 (LP69-2). The presence of polyphenols, in combination with PVA, improved the thermal stability, radical-scavenging activity, probiotic survival, and overall antioxidant performance of the nanofibers (Ma et al., 2024).

**Proteins** possess strong functional potential for encapsulating aromatic compounds owing to their amphiphilic structure, multiple binding sites, and various interaction mechanisms such as hydrogen and covalent bonding. Nevertheless, their complex secondary and tertiary conformations, along with poor electrospinning behavior of globular proteins, present significant processing challenges. These limitations may be mitigated by promoting random coil configurations through dissolution, cleavage of cysteine disulfide bonds, and the use of fluorinated solvents. Typical protein-based encapsulating materials include zein, whey protein concentrate, soy protein isolate, and fish sarcoplasmic proteins (Stoyanova et al., 2023). For instance, olive leaf polyphenols were successfully loaded into almond gum/gelatin electrospun nanofibers; the fiber diameter increased with higher polyphenol concentrations, and the release rate was initially high and continuous, remaining proportional throughout the process. (Geravand et al., 2025). Additionally, polylactic acid (PLA) nanofibers containing lavender essential oil were fabricated via electrospinning. These nanofibers exhibited nanometer-scale diameters, hydrophobic surfaces, and satisfactory thermal properties, indicating their suitability for food packaging and coating applications (Ismaili et al., 2024). Likewise, electrospun PLA/tea polyphenol (TP) nanofibrous membranes exhibited antibacterial activity, with clear inhibition of both Gram-positive (*Staphylococcus aureus*) and Gram-negative (*Escherichia coli*) strains (Wu et al., 2022).

#### 3.1.4 Safety, regulatory, and consumer considerations of electrospun food systems

Although many biopolymers used in electrospinning, including polysaccharides, proteins, lipids, and cellulose derivatives, are biodegradable and generally recognised as safe (GRAS), their safety in nanofibrous form requires further investigation. Regulatory approval of a polymer in its bulk form does not necessarily extend to electrospun nanofibers, because the electrospinning process may alter polymer conformation, crystallinity, surface area, and degradation behavior, potentially influencing digestibility, bioavailability, and interactions with biological systems. Since electrospun nanofibers may ultimately be incorporated into food matrices and consumed directly, the selected polymers should be food-grade, digestible, and free from adverse health effects. Despite the favorable safety profile of many food-grade polymers, limited information is available regarding the gastrointestinal fate, long-term toxicity, and potential cytotoxicity of electrospun nanostructures and their degradation products (da Silva and Silva, 2026). Beyond safety and regulatory considerations, the sensory impact and consumer perception of electrospun delivery systems are also critical factors determining their successful application in foods. Encapsulation within electrospun nanofibers can effectively mask undesirable tastes and odors associated with certain bioactive compounds and essential oils, thereby improving palatability without compromising functionality. Moreover, the controlled-release behavior of electrospun systems enables targeted delivery of aroma compounds during storage or consumption, thereby better preserving flavor quality and enhancing sensory performance. The incorporation of nanostructured materials may also improve product stability, appearance, and texture by promoting a more uniform distribution of active ingredients and reducing phase separation in complex food matrices. These advantages have the potential to increase consumer acceptance, particularly for functional foods enriched with health-promoting compounds (Otles et al., 2019).

### 3.2 Key parameters influencing electrospinning and Nanofiber properties

The electrospinning process is influenced by several parameters that can be broadly classified into three main categories: (1) polymer solution properties, (2) process parameters (machine-related), and (3) environmental conditions (Tahir et al., 2021). These parameters collectively affect the jet stability, morphology, and structural uniformity of the fibers, as well as functional properties such as encapsulation efficiency and release behavior (El-Hadi and Al-Jabri, 2016). A summary of the effects of electrospinning parameters on nanofiber properties is presented in Table 3. It shows that optimized parameters improve encapsulation efficiency and structural uniformity, and that even small changes in the process can significantly alter the release kinetics, indicating the sensitivity of electrospinning systems to these parameters.

**Table 3:** Effect of electrospinning parameters on nanofiber characteristics.

Parameter Category	Parameter	Effect on fiber morphology	Effect on encapsulation efficiency	Effect on release behavior	Practical limitation	References
**Polymer solution**	Molecular weight	↑chain entanglement →larger fiber diameter	↑ stability of loaded compounds	Slower release due to dense network	High viscosity limits spinnability	(Rajeev and Helms, 2023)
	Concentration	Bead-free fibers at optimal range; high concentration. → thicker fibers	↑ loading capacity	Sustained release	Excess concentration causes clogging	(Wu and Jin, 2008)
	Viscosity	High viscosity → thicker fibers	↑ retention of active compounds	Slower diffusion	Too high prevents jet formation	(Mousavi et al., 2023)
**Process**	Surface tension	Low surface tension → smooth, uniform fibers	↑ encapsulation stability	More controlled release	Requires surfactants/solvent optimization	Bhattarai et al. (2018)
	Conductivity	Higher conductivity → thinner fibers	↑ encapsulation via jet stretching	Faster release (higher surface area)	Excess salts may destabilize system	(Alkahtani et al., 2026; Bhattarai et al., 2018)
	Solvent volatility	Moderate volatility → uniform fibers	↑ efficiency of encapsulation	Tunable release	Too fast evaporation causes defects	(Omiyale et al., 2025; Zhang et al., 2019)
	Voltage	↑ voltage → thinner fibers (up to optimum)	May enhance encapsulation	Faster release due to small diameter	Jet instability at high voltage	(Akhgari et al., 2016; Bakar et al. (2019); Sun et al. (2019)
	Flow rate	High flow → thicker fibers, bead formation risk	↓ efficiency	Slower release	Incomplete solvent evaporation	Omiyale et al. (2025)
	Needle-to-collector distance	Controls drying time and fiber uniformity	↑ efficiency at optimal range	Tunable release	Too short → wet fibers	(Dzierzkowska and Stodolak-Zych, 2019; SalehHudin et al., 2018)
**Environment**	Needle diameter	Smaller needle → finer fibers	Slight ↑ efficiency	Faster release	Clogging risk	(Akhgari et al., 2016; Omiyale et al., 2025)
	Collector type	Rotating → aligned fibers	↑ uniform distribution	Directional release behavior	Equipment complexity	(Dzierzkowska and Stodolak-Zych, 2019; Sun et al., 2019)
	Temperature	↑ temperature → thinner fibers	↑ efficiency	Faster release	Thermal instability	(Abdulhussain et al., 2023; Dzierzkowska and Stodolak-Zych, 2019)
	Humidity	High humidity → porous/thicker fibers	↓ stability	Burst release tendency	Structural defects	(Dzierzkowska and Stodolak-Zych, 2019; Omiyale et al., 2025)
	Air pressure	Instability reduces uniformity	↓ encapsulation consistency	Irregular release	Hard to control conditions	(Dzierzkowska and Stodolak-Zych, 2019; Parajuli et al., 2016)

*The effects on encapsulation efficiency and release behavior are largely mediated through changes in fiber diameter, porosity, surface area, and structural integrity.

#### 3.2.1 Polymer solution properties

The properties of the polymer solution play a fundamental role in determining the electrospinning behavior and the resulting fiber structure. Molecular weight, concentration, viscosity, conductivity, surface tension, and solvent properties are key factors. Higher molecular weight polymers generally increase chain entanglement, leading to stable jet formation and larger fiber diameters. Similarly, increasing polymer concentration increases solution viscosity, resulting in thicker fibers; however, excessively low concentrations may lead to bead formation instead of continuous fibers (Rajeev and Helms, 2023). Viscosity, which is closely related to molecular weight and concentration, directly affects jet spinnability and fiber uniformity. Higher viscosity solutions yield thicker fibers, while lower viscosity yields thinner fibers; too low a viscosity prevents the formation of continuous fibers (Mousavi et al., 2023). Surface tension is another important factor, as fiber formation requires electrostatic forces to overcome the adhesive forces in solution. Lower surface tension facilitates the formation of smoother fibers and reduces bead defects. This can be achieved through solvent selection or the addition of surfactants (Bhattarai et al., 2018). Solution conductivity significantly influences the extent of charge carried by the jet. Higher conductivity enhances elongation forces, resulting in finer fibers and improved uniformity. Conductivity can be adjusted by incorporating ionic salts such as sodium phosphate, potassium phosphate, lithium chloride, or ammonium chloride (Bhattarai et al., 2018). Solvent properties, including volatility and evaporation rate, also play an important role. Highly volatile solvents cause rapid drying and thinning of fibers and may also lead to porosity or structural defects (Omiyale et al., 2025). In addition to their effects on fiber morphology, the properties of the polymer solution strongly influence the encapsulation efficiency and release kinetics. Higher polymer concentration, molecular weight, and viscosity typically result in denser fibrous matrices, thereby reducing the release rate and increasing the sustained release of encapsulated bioactive compounds (Matei et al., 2026).

#### 3.2.2 Process parameters (machine-related)

Operational parameters significantly influence fiber formation, jet dynamics, and morphological characteristics. These include applied voltage, flow rate, tip-to-collector distance, needle diameter, and collector type. Applied voltage is the driving force for jet initiation. At optimal levels, it overcomes surface tension and forms a stable Taylor cone. Increasing voltage generally reduces fiber diameter due to stronger electrostatic stretching; however, excessively high voltage may induce jet instability and bead formation (Bakar et al., 2019; Sun et al., 2019). The flow rate determines the volume of polymer solution delivered to the needle tip. Higher flow rates often result in thicker fibers due to insufficient time for solvent evaporation, while lower flow rates produce more uniform and finer fibers (Omiyale et al., 2025). The needle-to-collector distance should be sufficient to allow adequate solvent evaporation during jet travel. A short distance can result in thicker, bead-like, or irregular fibers due to incomplete solvent evaporation, whereas a longer distance promotes the formation of thinner fibers due to increased evaporation time. Therefore, this parameter must be optimized to ensure proper solvent removal and uniform fiber deposition on the collector (SalehHudin et al., 2018). Needle diameter influences the initial jet size; smaller needles generally produce finer fibers (Omiyale et al., 2025). Additionally, collector type affects fiber alignment: rotating collectors promote oriented fiber structures, while static collectors yield randomly distributed fibers (Sun et al., 2019). Process parameters indirectly affect release behavior by influencing fiber diameter, porosity, and structural uniformity. For example, thinner fibers produced under optimized voltage and flow rate conditions typically exhibit a larger surface area-to-volume ratio, which may accelerate the release of encapsulated compounds (Sumathi, 2025).

#### 3.2.3 Environmental conditions

Environmental factors such as temperature, humidity, and air pressure also significantly affect electrospinning results. Higher temperatures reduce solution viscosity and accelerate solvent evaporation, typically resulting in thinner fibers. However, excessive temperature near solvent boiling points may induce pore formation due to rapid phase separation (Abdulhussain et al., 2023). Relative humidity strongly affects the morphology and porosity of fibers. High humidity slows solvent evaporation, often leading to thicker fibers and increased porosity due to water vapor condensation and phase separation (Omiyale et al., 2025). Although electrospinning is typically conducted at atmospheric pressure, fluctuations in air pressure can affect jet stability and deposition patterns, ultimately influencing fiber uniformity (Parajuli et al., 2016). Environmental conditions can also influence release performance by altering fiber porosity and internal structure. Increased humidity often promotes pore formation, which may facilitate faster diffusion of active compounds and contribute to an initial burst release effect (Ramos et al., 2021).

### 3.3 Controlled release of aromatic plant compounds via electrospinning technology

In the electrospinning process, factors such as polymer composition, environmental conditions, and compound volatility can facilitate the encapsulation of volatile compounds via functionalization, thereby enabling controlled, prolonged release (Partheniadis et al., 2020). Accordingly, as shown in Table 3, factors such as temperature, humidity, and matrix polarity accelerate diffusion and evaporation, whereas polymer-aromatic compound interactions may delay release. Therefore, the design of effective carrier systems increases based on the intended food application. Stimulus-responsive electrospun systems have recently been used as an advanced strategy for the controlled release of aromatic and bioactive compounds in food products. These systems are designed to respond to external or internal environmental stimuli and exhibit targeted release behavior rather than passive diffusion. These responses are usually achieved through physicochemical changes in the polymer matrix, including swelling, degradation, structural changes, or disruption of noncovalent interactions, which collectively alter the fiber porosity and release pathways. This can significantly affect the stability and release of encapsulated aromatic compounds in complex food matrices where temperature, humidity, and pH fluctuate (Min et al., 2021). Despite these advantages, several challenges limit their practical application. The reactivity of these systems depends on the intensity, duration, and uniformity of external stimuli, which may result in incomplete or uncontrolled release under real-food conditions. Furthermore, the fabrication of multi-reactive systems often requires complex polymer designs, specialized materials, and multi-step processing, which can increase production costs and reduce scalability. Also, variability in food environments may undermine reproducibility and performance stability, especially in large-scale applications. In addition to these conventional triggers, enzyme-responsive systems have recently gained attention, particularly in food preservation applications, where spoilage-related enzymes secreted by microorganisms can act as natural biological triggers (Gao et al., 2025). This approach enables highly selective release behavior that is directly related to food spoilage processes and offers a promising avenue for food preservation systems. Accordingly, Table 4 indicates that the controlled release of aromatic compounds is affected by factors such as temperature, humidity, pH, and light. Among these factors, temperature and humidity, through thermal effects and swelling, increase matrix permeability and accelerate the release of aromatic compounds. In addition, light and pH can affect the release pattern by changing and degrading them. Therefore, environmental stimuli, by altering the physicochemical properties of electrospun nanofibers, create a porous structure that enables more stable, targeted release of aromatic compounds than conventional encapsulation methods. Controlled release of essential oils from nanofibers can also reduce the cytotoxic effects of certain essential oil components on human cells (Rather et al., 2021). Bioactive constituents of essential oils are prone to degradation under several conditions, such as variations in pH, oxygen exposure, elevated temperatures, light exposure, and other environmental factors (Lee et al., 2016; Hurley et al., 2021). Therefore, the encapsulation of essential oils should be carefully controlled to mitigate the effects of these degradative factors and preserve the activity of their key bioactive constituents, thereby improving shelf life in food systems. In this context, selecting an appropriate controlled-release system is critical to ensuring the stability and functionality of essential oils (Froiio et al., 2019; Yi et al., 2022).

**Table 4:** Factors affecting the controlled release of aromatic compounds.

Stimulus	Release Mechanism	Encapsulation Material/Polymers	Active Compounds	Stimulation Conditions	Reference
Light	Essential oil release by changing the molecular structure of a light-sensitive polymer	Synthetic or natural light-sensitive polymers, photochromic polymers such as azobenzene	Basil essential oil, thyme, orange terpenes	UV light irradiation (*λ*_max_ = 360 nm)	Marturano et al. (2018)
pH	Swelling or degradation of fiber structure in response to pH changes	Polyacids, polybasics, biopolymers, industrial polypeptides such as chitosan	Various essential oils	Acidic or alkaline environment with different pH limits	Ahmed and Mohmmed (2025)
Temperature	Increasing evaporation by converting thermal energy into kinetic energy in essential oils	Temperature-sensitive polymer materials (N- isopropylacryla mide)	Lemon essential oil	Temperature increase of about (∼32 °C)	(Abdelhameed et al., 2021; Xu et al., 2024)
Humidity	Facilitating the release of essential oils by swelling and increasing the porosity of moisture-absorbing matrices due to changes in ambient humidity.	graphene oxide, metal oxides, polymers	Thyme essential oil	Change in relative humidity (20-80%)	Min et al. (2021)

Studies on the sustained release and control of essential oil compounds in recent years have focused on the factors that stimulate their release. In the following, the most important of these factors are briefly reviewed.

1) Light: In photoresponsive electrospun systems, exposure to light induces photoisomerization, photocleavage, or photothermal effects within the polymer matrix. These processes alter polymer chain mobility and may lead to chain rearrangement, expansion of free volume, or partial disruption of the fiber network, thereby increasing porosity and facilitating the diffusion of encapsulated compounds. In core-shell fibers, light-induced structural changes can also modify interfacial permeability between the core and shell layers, enabling triggered release of aromatic compounds (Marturano et al., 2018).
2) pH: In this method, polymers that respond to pH changes should be used and used for encapsulation, such as polyacids, polybases, biopolymers, industrial polypeptides and polymers that are degradable to pH changes, the most common of which in the encapsulation industry is chitosan, whose amino group has alkaline properties and can produce ammonium salts in acidic environments, So pH-responsive polymers undergo ionization or protonation–deprotonation reactions in response to environmental pH changes. These reactions alter electrostatic interactions between polymer chains, leading to swelling, contraction, or partial degradation of the polymer matrix. Consequently, pore size and diffusion pathways are modified, significantly affecting the diffusion coefficient and release kinetics of encapsulated compounds. In core-shell systems, pH-induced swelling may weaken interfacial interactions and accelerate the migration of volatile compounds from the core region (H. A. M. Ahmed and Mohmmed, 2025).
3) Temperature: This method requires the use of encapsulating materials that can convert the applied thermal and thermal energy into kinetic energy in the molecules of essential oils (especially volatile compounds such as aromatic oils) and cause the controlled release of the compounds (Abdelhameed et al., 2021). These systems rely on thermally induced changes in polymer chain mobility and molecular relaxation. Increasing temperature enhances chain flexibility and free volume within the matrix, increases diffusion coefficients, and may induce phase transitions or structural rearrangements in certain polymers. For volatile aromatic compounds, elevated temperatures also increase vapor pressure, thereby promoting diffusion through the polymer network. In core-shell structures, thermal expansion or softening of the shell layer can facilitate the release of encapsulated compounds (Abdelhameed et al., 2021).
4) Humidity: In this case, encapsulating materials that respond to changes in humidity, such as graphene oxide, metal oxides, polymers, etc., have been used, which allow for the controlled release of the effective compounds of essential oils under controlled humidity conditions. Moisture-responsive polymers absorb water molecules that act as plasticizers, reducing intermolecular interactions and increasing polymer chain mobility. Water uptake often induces swelling and pore enlargement, thereby enhancing mass transfer and diffusion of encapsulated compounds. In multilayer and core-shell systems, differential water absorption between the shell and core materials may alter interfacial integrity and generate new diffusion pathways, resulting in controlled release under humid conditions (Min et al., 2021).

## 4 Migration and controlled release of bioactive aromatic compounds in food systems

The migration of bioactive aromatic compounds from delivery systems into food matrices plays a crucial role in their functional efficacy, especially in antimicrobial and antioxidant activities. For this reason, the development of controlled-release systems, such as encapsulation and electrospun nanofibers, has attracted attention to regulate this migration behavior and maintain compound stability during the storage period of food products. These systems should be designed so that, in addition to preventing rapid release, they also prevent rapid degradation of the active compounds. Fig. 2 shows how aromatic compounds are released in encapsulation and electrospinning systems according to the mechanisms of diffusion, degradation of the carrier matrix, or a combination of both. In conventional methods, micro/nanocapsules undergo swelling, rupture, or loss of wall material, resulting in rapid, uncontrolled release, whereas electrospun nanofibers provide more controllable, sustained release over time due to their high surface area, structural uniformity, and morphology (Tarahi et al., 2026). The type of encapsulating material, including liposomes, nanoemulsions, cyclodextrins, and biopolymer nanoparticles, plays an important role in how active compounds interact with the matrix, allowing them to be physically trapped or attached to the carrier through chemical interactions (Rezagholizade-Shirvan et al., 2024). In these systems, the concentration gradient between the carrier and the food substance is often the main factor determining the release rate. In addition, environmental conditions such as temperature, pH, water activity, and the chemical composition of the food (especially fat and protein content) significantly affect compound migration (Shiravani et al., 2024). In contrast, electrospun nanofibers, owing to their high surface area-to-volume ratio and tunable structure, offer an advanced platform for the controlled release of aromatic compounds, particularly volatile and sensitive ones (Mathew et al., 2025). In these systems, bioactive compounds can be incorporated into the fiber structure through methods such as direct mixing, emulsion electrospinning, or coaxial electrospinning, each of which yields distinct release patterns (Lian et al., 2024). The migration mechanism in these fibers usually involves diffusion, swelling, or relaxation of polymer chains, and in some cases, destruction of the fiber structure (de Farias et al., 2025). Fig. 3 schematically shows the migration path of aromatic compounds after release from capsules or nanofibers into food matrices. These compounds can migrate through diffusion or diffusion into the lipid phase, absorption into the food component or evaporation from the upper surface. Migration mechanisms, influenced by environmental factors such as temperature, humidity, storage time, and pH, play an important role in preserving flavor, sensory perception, and the stability of food products. Compared with conventional encapsulation systems, electrospun nanofibers often provide more controlled, sustained release and minimize sudden release (Sumathi, 2025). One of the key differences between these two approaches is how they interact with the food environment. Encapsulated systems typically exhibit faster release upon contact with aqueous or lipid phases, depending on the solubility of the active ingredients (Xu et al., 2024). In contrast, electrospun nanofibers can serve as part of active packaging, allowing the gradual migration of ingredients into the food and its packaging (Mathew et al., 2025). This feature makes them well-suited for applications aimed at extending the long-term shelf life of foods. On the other hand, the physicochemical properties of bioactive compounds also directly influence migration behavior; factors such as molecular weight, volatility, and hydrophobicity determine the affinity of compounds for different food phases (Pérez-Pérez et al., 2024). For example, compounds such as thymol, carvacrol, and eugenol have a higher affinity for fatty phases, which can increase their migration in high-fat foods but may also limit their availability in aqueous systems (Pérez-Pérez et al., 2024). In controlled release systems, the transport of aromatic compounds is influenced by various mechanisms, and several mathematical models are used to describe the release behavior of bioactive compounds.

1) *Zero-order* model: represents a steady-state release profile under ideal conditions, i.e., the release kinetics of an agent occur at a constant rate over time and are independent of the concentration of the active compound (L. Ahmed et al., 2019).
2) *First-order* model: the release rate depends on the concentration of the active compound remaining in the carrier over time, meaning that with a decreasing amount of active compound, the release rate gradually decreases with time, and is often applied to aromatic compounds encapsulated in biodegradable matrices (Heredia et al., 2022).
3) *Higuchi* model: release from a polymer matrix is a diffusion-based process governed by Fick's law, in which the amount of active agent released is proportional to the square root of time (∝ √t). Its application is limited to systems that do not exhibit significant swelling, relaxation, or degradation of the polymer matrix (Viswanadha et al., 2024).
4) *Korsmeyer–Peppas* model: It is one of the most widely used models for evaluating the release kinetics of aromatic compounds. The release mechanism includes both Fickian and non-Fickian behaviors and is described by the release exponent (n), as follows:
  i. For n < 0.45, it represents quasi-Fickian diffusion.
  ii. When n = 0.45, it denotes Fickian diffusion.
  iii. In the range 0.45 < n < 0.89, it signifies non-Fickian (anomalous) diffusion characterized by a combination of diffusion and polymer relaxation.
  iv. When n ≥ 0.89, it corresponds to Case II transport (gel swelling) (Werzer et al., 2019).

**Fig. 3:**
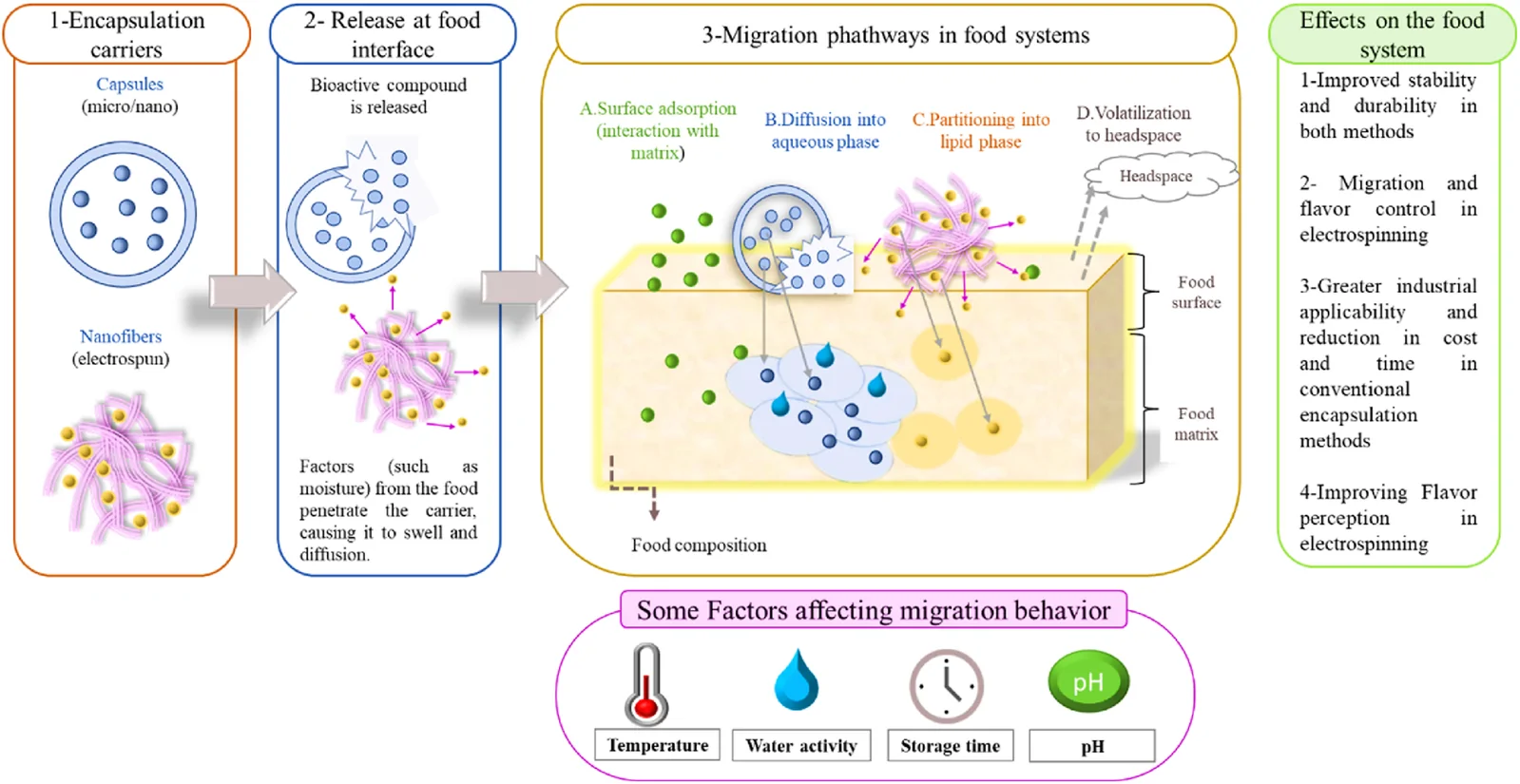
Visual analysis of the migration process of bioactive flavoring compounds in food systems: The role of capsules and nanofibers.

Fickian diffusion occurs when the release is mainly controlled by the concentration gradient within the carrier matrix. In contrast, non-Fickian or anomalous transport results from the simultaneous effect of diffusion and relaxation of the polymer chains. In biodegradable carriers, matrix degradation or erosion can also play an important role in compound release, a phenomenon known as erosion-controlled release. The *Ritger–Peppas* model is widely used for polymer systems with mixed release mechanisms and is commonly used to distinguish between diffusion-controlled release and release due to matrix swelling or erosion (Kou et al., 2025). Although these mathematical models are widely used to describe release behavior, it is important to note that the release of aromatic compounds from encapsulation systems and electrospun nanofibers often occurs through several simultaneous mechanisms rather than a single ideal process. As a result, simplified kinetic models such as *zero-order*, *first-order*, or even *Higuchi* may not fully capture the complexity of release behavior in real food and packaging systems. Among the available models, the *Ritger–Peppas* and *Korsmeyer–Peppas* models have been most widely reported in recent studies on essential oils and electrospun nanofibers, as they can describe non-Fickian and anomalous transport behavior via the release exponent (n).

Therefore, the optimal design of the carrier system according to the type of food is essential to achieve the desired release kinetics. A bilayer active film consisting of Persian acorn starch and zein nanofibers, loaded with 10% (v/w) cinnamon essential oil (CEO), was fabricated via casting and electrospinning. The resulting acorn starch/zein/CEO film reduced lipid oxidation by 44%, inhibited total mesophilic bacterial growth, and extended the shelf life of rainbow trout fillets by 6 days. These effects were attributed to the controlled, slow release of CEO, which enhanced the film's antibacterial and antioxidant properties (Kazemi et al., 2026). A smart composite pad was fabricated by encapsulating cinnamon–oregano essential oil within gelatin–chitosan microcapsules (COEOMs) and embedding them in a polyvinyl alcohol/konjac glucomannan (PK) matrix, thereby forming antimicrobial PK-COEOM pads. The system was designed to enable gelatinase-triggered controlled release, with emphasis on the release kinetics of the encapsulated essential oil. The Ritger–Peppas model indicated that COEO release was predominantly controlled by Fickian diffusion and was influenced by both concentration and temperature. Furthermore, the application to chilled pork preservation revealed synergistic antimicrobial effects via direct contact and vapor-phase inhibition, resulting in a shelf-life extension of at least 8 days at 4 °C (Gao et al., 2025).

These studies show that the release behavior and effectiveness of electrospun systems depend on the interplay among polymer characteristics, bioactive compound properties, and the composition of the food matrix (Coelho et al., 2021; Falsafi et al., 2023; Rostamabadi et al., 2020; Wang et al., 2023). The nature of the encapsulated bioactive compound is particularly important because, to minimize the loss of highly volatile aromatic compounds, the carrier matrix should interact strongly with these compounds and effectively entrap them within its structure (Castro-Muñoz et al., 2023; Falsafi et al., 2023). Furthermore, the composition of the food matrix significantly affects the migration and release behavior of active compounds (Coelho et al., 2021; Falsafi et al., 2023; Wang et al., 2023). Hydrophilic matrices, such as starch- and gelatin-based films, generally exhibit controlled release and faster diffusion over a shorter period due to their greater water absorption and swelling capacity (Castro-Muñoz et al., 2023). In contrast, hydrophobic or multilayer matrices, such as zein nanofibers, PVA/QAC, and rosin-based systems, reduce evaporation and regulate the vapor phase of essential oils, thereby preventing rapid diffusion and promoting a slower, more sustained release of volatile aromatic compounds (Castro-Muñoz et al., 2023; Falsafi et al., 2023). On the other hand, stimulus-responsive systems, such as gelatinase-sensitive PK-COEOM pads, represent a more advanced approach in which the release of active compounds is triggered by microbial activity (Table 1). Also, in terms of food matrix characteristics, lipid-rich foods, such as fish, interact favorably with hydrophobic compounds, including thymol, carvacrol, cinnamaldehyde, and other essential oils. Through the prolonged release of antioxidant and antimicrobial compounds, these systems effectively delay lipid oxidation and microbial spoilage (Falsafi et al., 2023). In contrast, high-moisture foods generally accelerate the diffusion and release of active compounds (Rostamabadi et al., 2020; Wang et al., 2023). Protein-rich matrices, such as pork, are more suitable for enzyme-responsive systems because microbial enzymes produced during spoilage can accelerate the targeted release of active compounds, thereby enhancing product shelf life. Although most studies to date have been conducted under laboratory conditions, further studies on large-scale production, long-term stability, and economic and regulatory aspects are still required before their widespread industrial implementation can be achieved (Falsafi et al., 2023).

## 5 Comparison of classical encapsulation with electrospinning

In general, each conventional and electrospinning encapsulation method shown in Table 5 differs in terms of encapsulation efficiency, release behavior, sizes, and reagents, which make it selected based on the type of aromatic compound and the intended application. Spray drying encapsulation is a well-known technique that provides controlled solubility and release of aromatic compounds in powder form, helping to reduce storage and transportation costs by helping to stabilize microbiological products. As shown in Table 5, the comparison between conventional encapsulation methods and electrospinning indicates that the selection of an encapsulation technology should be application-specific rather than based solely on encapsulation efficiency. Electrospinning offers advantages in terms of controlled release, protection of volatile compounds, and the production of nano-scale fibers, making it particularly suitable for high-value functional ingredients and advanced delivery systems. However, conventional techniques remain preferable when large production volumes, established manufacturing infrastructure, regulatory familiarity, and lower processing costs are the primary requirements. Therefore, electrospinning should be viewed as a complementary technology that expands the range of encapsulation strategies rather than a universal replacement for conventional industrial methods. However, this method has two major limitations: 1) the need to use a film-forming carrier that is soluble in water and 2) the requirement that the active ingredients be heat-resistant, as the process involves exposure to high temperatures. Although spray drying is more widely used in industry than freeze drying and electrospinning to produce powders containing aromatic compounds, its encapsulation efficiency is often reduced due to the lack of precise control over the final particle properties, such as size distribution, morphology, damage to volatile compounds, as well as process parameters (Coelho et al., 2021). In contrast, spray cooling and freeze drying are considered more suitable techniques for encapsulating heat-sensitive aromatic compounds that are unstable in aqueous systems, and the encapsulated products have better appearance, color, taste, and aroma. However, it has been reported to be less efficient than electrospinning (Golpira et al., 2021). For example, Golpira et al. (2021) encapsulated saffron extract in a gelatin matrix using both electrospinning and freeze-drying methods and compared their performance. The results showed that the electrospun fibers and particles containing the extract had smooth morphology, homogeneous distribution, higher encapsulation efficiency (74%), and better thermal stability, while the particles produced by freeze-drying showed larger and irregular structures with lower encapsulation efficiency (65%), highlighting the potential of electrospinning as an encapsulation technique for sensitive bioactive compounds. On the other hand, direct comparative studies between coacervation and electrospinning for aromatic compounds are limited, so examining the results reported for a specific aromatic compound can allow for an indirect comparison of the two methods. Accordingly, in a study, the aromatic compound thymol from ovion was used for encapsulation by the coacervation method. The results showed that using polysaccharide wall materials, microcapsules of about 5-50 μm were formed, and the encapsulation efficiency was 65%, which led to a slower release and increased thermal stability of the aromatic compound (Chaib et al., 2021). Therefore, this method is suitable for industrial applications that require longer control with slower release of compounds. On the other hand, a study in line with this material but using the electrospinning method was presented by Ali et al. (2024), in which thymol from ovion was placed in the structure of pullulan/whey composite nanofibers. According to these results, the formed nanofibers were uniform, had an encapsulation efficiency of 87%, could enable controlled release in the small intestinal environment, and exhibited antioxidant properties. Therefore, the electrospinning-based nanofiber system can enable the use of aromatic compounds in the food and pharmaceutical industries through controlled transport and release. By comparing these two methods for aromatic compounds, it can be concluded that the coacervation method is mainly suitable for producing microcapsules with an efficiency of about 60 to 70% and specific wall materials, and electrospinning, with higher efficiency (∼90%), has received more attention for the development of industrial and performance-oriented systems. All in all, spray drying, freeze-drying, and coacervation methods offer different performance characteristics depending on the encapsulated aromatic compounds, wall materials, and application requirements. In summary, spray drying has high industrialization potential and low operating cost, but is limited in the selection of wall materials and core compositions. Freeze-drying is suitable for heat-sensitive aromatic compounds, but it has high energy consumption and requires longer processing times. Coacervation produces high encapsulation efficiency and a more spherical structure, but requires precise control of formulation conditions and the interaction of wall and core materials. However, electrospinning produces a more uniform structure, greater tunability of release, and protection of volatile and heat-sensitive aromatic compounds compared to the aforementioned encapsulation methods, and it can emerge as a promising alternative. Yet limitations such as low throughput, solvent selection, and poor scalability still pose challenges for industrial adoption.

**Table 5:** Advantages and disadvantages of the most common techniques and electrospinning.

Encapsulation Technique	Advantages	Advantages	Disadvantages	Disadvantages	Particle size (μm)	Appearance of particles	Encapsulation efficiency (EE%)	Release behavior	Reference
Spray drying	- Simple method with short production time - Medium encapsulation efficiency - Scalable to industrial production, compatible with food ingredients - Low cost, high industrial application - Uniform and spherical structures - Microbiological stability of the produced product	- High temperature process - Unsuitable for heat-sensitive compounds - Adhesion of active ingredient particles to the drying chamber	1-150	Spherical, smooth, and slightly brittle	87-90%	Fast	(Laina et al., 2024; Nguyen et al., 2021)		
Spray chilling	- Low temperature - Low cost - Use of edible lipids in the process	- Changes in composition due to the rapid cooling rate - Low encapsulation efficiency - Low stability - low industrial application	2-200	Spherical and hard	85-90% (Depending on the lipid carrier)	Moderate	Favaro-Trindade et al. (2021)		
Freeze-drying	- Long-term storage - Large-scale production - Suitable for heat-sensitive food and pharmaceutical compounds - Retention of color, shape, and aroma of encapsulated compounds	- Complex process - High cost, low industrial application - Limitations in the use of wall materials due to solubility in aqueous solution - Relatively low encapsulation efficiency	1-100	Porous and spongy	66-70%	Moderate	(Laina et al., 2024; Rezvankhah et al., 2020)		
Complex coacervation	- Suitable for temperature-sensitive compounds - Process at room temperature - Low cost - Uses low concentrations of surfactants - Can be performed in aqueous and non-aqueous environments - Use of edible biopolymers	- Use of toxic chemical solvents - Solvent residue in the structure - Agglomeration of droplets - limited industrial application	5-200	Spherical, smooth surface	73-75%	Controlled	(Chaib et al., 2021; Evcin et al., 2024)		
Electrospinning	- Simple and versatile techniques - High encapsulation efficiency - Increases stability of aromatic compounds - Suitable for temperature-sensitive compounds - Process at room temperature - No use of toxic chemicals - Controls the release of aromatic compounds in food - Emerging and developable in the industry	- Difficult to scale up -Not all wall materials can be used due to viscoelastic conditions - Limitations in the choice of food-grade solvents in some formulations	0/1-1 (nm-μm)	Thin nanofibers (fiber-like)	85-90%	Slowly and systematically	(Coelho et al., 2021; Lian et al., 2024)		

## 6 Flavor application in food industries

The development of industrial food products has led to the creation of foods with novel characteristics and enabled the processing of functional foods with increasingly complex properties through the application of advanced techniques such as encapsulation (Wang et al., 2023). Flavor application plays a central role in the food industry and in flavor manufacturing companies. In addition to developing new flavors, these companies are involved in product formulation and performance evaluation of flavors in health-oriented products designed to meet consumer needs in disease prevention (Sri Padma and Joshi, 2023). Encapsulated flavors are widely used in a range of food products, including snacks, beverages, and dairy formulations. In soft drinks, orange, lemon, and cola are among the most commonly used flavoring agents. Hydrophobic flavor oils such as orange, lemon, and mint are typically dispersed in aqueous systems as colloidal emulsions prior to incorporation. These flavor compounds are often delivered as encapsulated emulsions prepared using appropriate encapsulation techniques. One of the primary criteria for evaluating encapsulation efficiency in food systems is its ability to maintain flavor stability while enabling controlled release (Mohebbi, 2020). Recent studies have demonstrated the potential of electrospinning as an effective approach for improving the delivery and stabilization of bioactive molecules, thereby protecting functional compounds in food matrices while ensuring flavor stability and desirable sensory attributes in the final products (Mohebbi, 2020). The bioactive plant extracts, when used as functional ingredients, often face challenges related to direct incorporation into food systems, such as instability or poor solubility. Electrospinning overcomes these limitations by producing fibers that offer high release efficiency, enhanced stability, and preservation of compound properties without compromising fiber morphology. Moreover, researchers have reported that electrospun fibers significantly improve the dissolution rate of poorly water-soluble compounds, such as oil-based extracts, due to their high porosity and large surface area-to-mass ratio. These characteristics make electrospinning a promising encapsulation technology for the food industry, particularly for developing functional foods with stable, controlled flavor release (Wang et al., 2023).

### 6.1 Application of electrospun fibers containing volatile aromatic compounds in the food industry

Electrospun nanofibers have a wide range of applications. The incorporation of essential oils (EOs) as antimicrobial and antioxidant agents into active food packaging through electrospinning is an emerging strategy to prolong shelf life and preserve food safety, quality, and health attributes. As can be seen in Table 4, this approach improves the stability of volatile compounds, reduces the intensity of essential oil flavors, and facilitates controlled release (Ataei et al., 2020). Göksen et al. (2021) developed an active packaging system using electrospun polyvinyl alcohol (PVA) nanofibers loaded with essential oils from *Laurus nobilis* (LEO) and *Rosmarinus officinalis* (REO) to enhance the shelf life of chicken breast fillets. The system reduced lipid oxidation by up to 68%, demonstrated antimicrobial activity, and improved pH and color stability during storage. In another study, Vafania et al. (2019) synthesized gelatin/chitosan nanofibers loaded with thyme essential oil using a nozzleless electrospinning technique. The antioxidant and antimicrobial properties of the developed nanofibers, as well as their effectiveness in reducing nitrite levels in sausages, were investigated. The findings revealed that encapsulated thyme essential oil exhibited bactericidal activity against *Clostridium perfringens*, compared with samples containing 120 ppm nitrite, without adversely affecting the color or sensory properties of the sausages. Overall, the results suggest that these nanofibers may represent a viable alternative to nitrite in meat product applications. Doğan et al. (2022) investigated the use of lavender essential oil (LEO)-loaded nanofibers for the preservation of minced lamb and reported reduced growth of aerobic mesophilic bacteria, psychrophilic bacteria, molds, and yeasts, resulting in an extended shelf life. Similarly, Bahramian et al. (2025) developed electrospun polycaprolactone (PCL) nanofibers incorporating lemon essential oil (*Aloysia citriodora*) and chromium (III)-metal-organic framework (Cr(III)-MOF) for active packaging of red meat stored at 4 °C. These nanofibers showed antibacterial activity against *Staphylococcus aureus* and *E. coli* and effectively maintained chemical (TBA, pH, TVB-N, PV) and microbial (PTC, TVC) quality indicators, extending shelf life by up to 18 days. In addition, electrospun nanofibers containing grape seed oil (gsN) were applied as a coating for fish meat under cold storage conditions, where they effectively delayed the growth of total aerobic mesophilic bacteria, molds, and yeasts, and exhibited strong antioxidant activity by inhibiting lipid oxidation in fish samples (Ceylan et al., 2021). Similarly, cinnamon essential oil/β-cyclodextrin inclusion complex (CEO/β-CD-IC) incorporated into polylactic acid (PLA) nanofibers via electrospinning showed antimicrobial activity against both Gram-positive (*Staphylococcus aureus*) and Gram-negative (*Escherichia coli*) bacteria. The antimicrobial mechanism of essential oils is primarily attributed to disruption of cell wall biosynthesis, membrane function, and enzymatic activity in microorganisms (Faikoh et al., 2014). Moreover, these electrospun fibers have been shown to effectively extend the shelf life of pork (Wen et al., 2016). The encapsulation of cinnamon essential oil/β-cyclodextrin proteoliposomes within polyethylene oxide nanofibers has demonstrated antimicrobial activity against *Bacillus cereus*, a key microorganism responsible for contamination and spoilage in meat products. When used as active packaging for beef, these nanofibers demonstrated high antibacterial efficacy, extending shelf life and maintaining the product's sensory quality (Lin et al., 2017).

## 7 Future perspectives

Future research should focus on a broader evaluation of the controlled release of flavors and their application in diverse food systems. In particular, further studies are needed to examine the combined effects of emerging processing technologies, such as electrospinning, with classical techniques including spray drying, freeze drying, and coacervation; however, the promising potential of electrospinning for flavor encapsulation, several scientific and technological challenges remain before its widespread implementation in food systems. First, most studies have been conducted in simplified model media, and the performance of electrospun flavor delivery systems in real food matrices, including dairy, meat, bakery, and beverage matrices, remains poorly understood. The complex composition of foods, including proteins, lipids, carbohydrates, salts, and variations in water activity, may substantially influence fiber integrity, encapsulation efficiency, aroma retention, and release kinetics. Therefore, future studies should validate electrospun systems in real food products and under realistic processing, storage, and consumption conditions. Second, predictive models that correlate fiber morphology, polymer characteristics, and environmental factors with flavor release kinetics remain limited. The integration of computational approaches, including mathematical modeling and machine learning algorithms, may facilitate the rational design and optimization of electrospun delivery systems and accelerate the development of tailored release profiles. Another major challenge is the commercialization and cost-effectiveness of electrospinning technology. Although high-throughput methods such as multi-needle and needleless electrospinning have significantly increased production rates, maintaining fiber uniformity, encapsulation efficiency, and process reproducibility at an industrial scale remains difficult. Further research is also required to develop solvent-free or greener electrospinning approaches that minimize residual solvent concerns and improve environmental sustainability.

In addition, regulatory and safety considerations require greater attention. Future investigations should evaluate the migration behavior of electrospun materials, the gastrointestinal fate and long-term toxicity of nanostructures, and their compliance with food-contact regulations. Finally, although electrospinning offers significant advantages for flavor protection and controlled release, limited information is available regarding sensory performance and consumer acceptance. Comprehensive sensory studies and consumer perception analyses are therefore needed to determine whether electrospun delivery systems can maintain desirable flavor characteristics and gain market acceptance in commercial food products.

## 8 Conclusion

This review indicates that the effectiveness of flavor encapsulation depends not only on encapsulation efficiency but also on the compatibility between flavor compounds, carrier materials, processing conditions, and the intended food application. Consequently, selecting a suitable encapsulation method and developing it for commercial applications requires a thorough understanding of the advantages and limitations of both conventional and emerging encapsulation techniques, taking into account the chemical properties of the flavor compounds and their intended applications in food systems. Conventional techniques such as spray drying, freeze drying, and coagulation have advantages depending on process conditions, cost, and industrial scale, but are limited by limited control over release profiles, non-uniform particle size distribution, and restrictions on wall material choices, which can reduce encapsulation efficiency and stability. In recent years, electrospinning has emerged as a promising alternative to conventional encapsulation methods. Electrospun nanofibers offer the ability to produce fibers with tunable properties at ambient temperatures, protect flavor compounds from heat, light, and chemical degradation, and enable controlled release at specific target sites in food systems. However, they face challenges such as low production throughput, limited solvent selection, and limited scalability. Future research should highlight the potential of electrospinning to develop novel, sustainable, and health-oriented food products and green solvent systems, and to investigate release behavior in food matrices to facilitate industrial and nutritional applications.

## Informed consent statement

Not applicable.

## Author contributions

Conceptualization, S.M.B.H and E.A; supervision, D.J., Z. S., E.A., and S.M.B.H; writing—original draft preparation, M.S; writing—review and editing. All authors have read and agreed to the published version of the manuscript.

## Institutional review board statement

Not applicable.

## Data availability statement

Data are available in the paper.

## Statement

The authors used AI for showing the mode of action.

## Funding

Not applicable.

## Declaration of competing interest

The authors declare that they have no known competing financial interests or personal relationships that could have appeared to influence the work reported in this paper.
